# The potato rhizosphere microbiota correlated to the yield of three different regions in Korea

**DOI:** 10.1038/s41598-024-55263-7

**Published:** 2024-02-24

**Authors:** Gye-Ryeong Bak, Kiseok Keith Lee, Ian M. Clark, Tim H. Mauchline, Vanessa Nessner Kavamura, George Lund, Samnyu Jee, Jeong-Tae Lee, Hyun Kim, Yong-Hwan Lee

**Affiliations:** 1 Highland Agriculture Research Institute, National Institute of Crop Science, Rural Development Administration, Pyeongchang, 25342 Republic of Korea; 2 Interdisciplinary Programs in Agricultural Genomics, Seoul National University, Seoul, 08826 Republic of Korea; 3 Department of Ecology and Evolution, The University of Chicago, 1101 East 57th Street, Chicago, IL 60637 USA; 4 Sustainable Agriculture Sciences, Rothamsted Research, Harpenden, AL5 2JQ Hertfordshire UK; 5 Department of Agricultural Biotechnology, Seoul National University, Seoul, 08826 Republic of Korea; 6 Research Institute of Agriculture and Life Sciences, Seoul National University, Seoul, 08826 Republic of Korea; 7 Center for Plant Microbiome Research, Seoul National University, Seoul, 08826 Republic of Korea; 8 Plant Immunity Research Center, Seoul National University, Seoul, 08826 Republic of Korea; 9 Plant Genomics and Breeding Institute, Seoul National University, Seoul, 08826 Republic of Korea

**Keywords:** Microbiome, Plant symbiosis

## Abstract

We examined potato rhizosphere bacterial and fungal communities across three regions: Cheongju, Pyeongchang, and Gangneung. These regions have varying soil and climate conditions, resulting in different yields. We found that precipitation was the main limiting factor in our study while soil physiochemical factors affect bacterial and fungal microbiota in correlation with yield. Both bacterial and fungal microbiota showed distinct patterns according to the regions. ASVs positively correlated with yield were predominantly found in the Pyeongchang region which also produced the highest yields, while ASVs negatively correlated with yield were associated with Gangneung where the lowest yields were observed. The greatest bacterial and fungal diversity was detected in Pyeongchang consisting of Propionibacteriales, Burkholderiales, and Vicinamibacteriales. Gangneung, on the other hand primarily belong to Sordariales, Mortierellales, Cystofilobasidiales, and Tremellales. The putative yield-negative ASVs detected in Gangneung may have been influenced by drought stress. This work has highlighted key bacterial and fungal taxa as well as core taxa that may potentially be associated with high and low yields of potato in relation to metadata which includes soil chemical and physical parameters as well as weather data. Taken together we suggest that this information can be used to assess site suitability for potato production.

## Introduction

Potato (*Solanum tuberosum* L.) is cultivated worldwide and is an important food crop providing basic nutritional requirements such as carbohydrates and dietary fibre and additional health benefits from antioxidants, vitamins, β-carotene, polyphenols, and minerals^1^. Potatoes are the sixth highest yielding crop worldwide, with 395 million tonnes harvested in 2020^2^. There are over 5000 potato varieties and many show different characteristics for disease resistance, tuber nutrient content, yield, environmental adaptations and growth period, so different cultivars are grown according to purpose, environment and country^3^. Potatoes for consumption are cultivated across Korea mainly during the spring and autumn season in plane fields while most seed potatoes are grown in the summer season in highland regions with the cultivar Sumi (Superior), used in this study, being a major cultivar in Korea grown mainly during the spring and summer season^4^.

Since the introduction of high-throughput sequencing methodologies many microbial community profile studies have been performed, relating crop plant microbiome status to crop production^5^ and have developed better understanding of plant–microbe interactions under different environmental conditions^5^. Research suggests that the microbiome associated with the host crop including the rhizosphere, phyllosphere, and endosphere play key roles in plant health and production^6^. The rhizosphere is the region of soil surrounding roots which is influenced by plant mucilage and root exudates and in which microbes form interactions with the host plants^7^. There are many factors including soil type, pH, environmental conditions of soil and climate, crop genetic factors and agricultural management (fertilizers, pesticides), cropping systems and tillage which affect microbial community structure^8^.

Previous research has been performed to elucidate the effect of different regions, cultivars, cropping systems, and developmental stages on potato rhizosphere microbiomes^9–11^. Furthermore, research on tuber storage time and microbiome development has also been reported^12^. Other studies have compared potato microbiomes under different agricultural amendments, including biocontrol strategies^13,14^. The results of these potato microbiome studies show consistency with those reported for other crops with niche^11,12,14,15^, region^9,16^, and agricultural management^10,13^ having significant effect on bulk soil, rhizosphere soil, and tuber microbiome profiles.

However, there is a dearth of studies which consider the importance of regional climate zones as well as soil physical and chemical characteristics on potato rhizosphere microbiomes selection and its relationship with potato yield. In addition, most potato microbiome research to date have concentrated mainly on bacterial community structure, with only a few considering fungal^11^ or both fungal and bacterial microbiomes^15,16^.

Hence, the purpose of this research is to identify the main bacterial and fungal populations associated with the potato rhizosphere (cv Sumi), their correlation with yield and the main environmental and agricultural drivers that affect these microbiomes across three different regions in Korea. This relationship will allow a better understanding of the management practices required to improve and maintain potato yields to be developed.

## Results and discussion

### Climate and soil conditions among three regions

The region of Pyeongchang showed the highest temperature and accumulated precipitation during the potato growing season of 2016, with Gangneung receiving the lowest precipitation, although it was exposed to the highest levels of solar radiation (Supplemental Table S1). The preferred climatic conditions for potato cultivation are a temperature range of between 14 and 23 °C and precipitation of between 300 and 450 mm^3^. The temperature and cumulative solar radiation of all three regions in 2016 were slightly higher than the 20-year average, although the temperatures of all regions were within the optimum range for potato cultivation. Cheongju and Gangneung, and Pyeongchang all received a lower amount of precipitation than the 20-year average with a 30%, 51% and 43% drop respectively, although for the latter this was still within the optimal range for potato cultivation. Temperature and precipitation are important factors for crop cultivation with researchers reporting that temperature has a more significant influence for potato crop yields^17^.

Incorporating precipitation data alongside soil moisture contents (Supplemental Fig. S3), Gangneung exhibited the lowest soil moisture contents during potato cultivation period, indicating nearly half of the moisture contents compared to other two regions from the flowering stage to harvest. Given that soil moisture contents play a crucial role in tuber bulking^4^, the low soil moisture contents during that period may have impacted potato yield in Gangneung.

The highest yields were obtained in Pyeongchang among the three regions (Supplemental Fig. S1). During the potato cropping season in 2016, it should be noted that there was no dominating disease pressure in any of the three regions. A large diurnal temperature range, within the optimum temperature range, has been shown to be positively correlated to potato yield^18^ and as Pyeongchang is at a higher altitude of 766 m, there is a larger diurnal temperature range when compared to the other regions. The higher temperature and greater diurnal temperature range at Pyeongchang may explain the higher yields obtained, although other factors may have contributed to this.

There were different soil conditions among the three chosen regions (Supplemental Fig. S2, Table 1). The results of soil texture analysis showed that a relatively low proportion of sand and higher proportions of both clay and silt were observed in Pyeongchang (classified as sandy clay loam) compared to the other two regions classified as sandy loam. Regarding soil chemical properties: pH, organic matter, available phosphate, potassium, calcium, and magnesium all showed significant differences among the three regions (Table 1). Cheongju recorded the highest pH and calcium values, although the lowest values for organic matter and potassium. Pyeongchang showed the highest value of magnesium and the lowest values for pH, available phosphate, and calcium. Gangneung showed the highest values for organic matter, available phosphate and potassium but the lowest value of magnesium.

**Table 1:** Soil chemical properties of three regions.

Region	pH (1:5)	Organic matter (g/kg)	Available phosphate (mg/kg)	K^+^	Ca^2+^	Mg^2+^
				(cmol/kg)		
Cheongju	7.8 ± 0.04^a^	31.7 ± 0.34^b^	790.7 ± 23.91^b^	0.6 ± 0.01^c^	10.3 ± 0.15^a^	2.3 ± 0.05^b^
Pyeongchang	6.1 ± 0.09^c^	45.0 ± 0.58^a^	299.7 ± 5.55^c^	0.8 ± 0.02^b^	4.3 ± 0.08^c^	3.7 ± 0.06^a^
Gangneung	6.5 ± 0.04^b^	46.7 ± 2.19^a^	1339.7 ± 18.42^a^	1.1 ± 0.02^a^	6.0 ± 0.08^b^	2.0 ± 0.02^c^
ANOVA	*	*	*	*	*	*

One-way analysis of variance (ANOVA) followed by test post hoc Fisher's LSD test was conducted to compare the mean values of each property among three regions. *Significance was determined at *p-value* < 0.05. Values are presented as mean ± standard error.

^a-c^Post hoc LSD analysis indicated significant differences (*p-value* < 0.05) among the regions.

According to the optimum soil nutritional requirements for potato cultivation in Korea, 20–30 g/kg of organic matter, 250–350 mg/kg of phosphate, 0.5–0.6 cmol/kg of potassium, 4.5–5.5 cmol/kg of calcium and 1.5–2.0 cmol/kg of magnesium were suggested^19^. Considering the above values, soil nutrient conditions across the three regions were all within these ranges, supplying the optimal levels or above of each nutrient although there were significant differences between the three regions.

### Comparisons of microbiota among the three regions

Microbial community profiling using Qiime2 of both bulk soil and rhizosphere samples identified a total of 10,139 bacterial ASVs and 1758 fungal ASVs. The bacterial rarefaction curves showed that the fewest ASVs were found in Gangneung (GN)—the region with the lowest potato yields (Supplemental Fig. S4A). In contrast, Pyeongchang (PC) which had the highest potato yields, had the least fungal ASVs (Supplemental Fig. S4B). The Venn diagrams also supported this result with the lowest bacterial ASV numbers in Gangneung and the lowest fungal ASV numbers in Pyeongchang (Supplemental Fig. S5).

PERMANOVA analysis and β-diversity showed that bacterial and fungal microbiota were significantly different between regions while there were no significant differences between niches within the same region for either bacterial or fungal microbiotas (Table 2, Supplemental Figs. S6, 7). When the PERMANOVA analysis was conducted separately for region and niche, it showed no significant differences in regions for bacterial rhizosphere soil. From the results of the Supplemental Fig. S6A, there was no clear distribution in rhizosphere soil compared to bulk soil. Some bacterial ASVs in the rhizosphere soil were strongly influenced by potato root and the Venn diagram results showed 3 times higher common bacterial ASVs in rhizosphere soil compared to bulk soils (Supplemental Fig. S5) which were consistent with the PERMANOVA and β-diversity results. Previous research has also demonstrated significant differences in microbial community structure between different regions and an association with different soil types^9,16^. There are few published papers on potato microbiomes, and most of them are focused on rhizosphere microbiota only, without comparison between bulk soil and rhizosphere soil. Although the effects of niche have been reported on for several crops including potato, maize, soybean, sorghum, and cotton, these previous studies reported no significant differences between the bulk and rhizosphere soil or only showed significant differences in the bacterial community but not in the fungal community^20–24^. In this study, bulk soils were collected between potato plants at a depth, so that they could be affected by the potato root. This might be one explanation for no significant differences between bulk and rhizosphere ASVs. Alternatively as the fields chosen for this study have had potatoes grown in them over many years, the selective pressure of potato cultivation over this extended period of time, may build up so that the bulk soil is more representative of a potato rhizosphere. A continuous legacy affect, which would not be so pronounced under crop rotations practices.

**Table 2:** PERMANOVA analysis of bacterial and fungal communities among three regions.

Domain	Bacteria		Fungi							
Treatment	Region	Niche	Region	Niche						
*d.f*	2	1	2	1						
*SS*	1.8781	0.4168	2.6843	0.2669						
*MS*	0.93903	0.41680	1.34217	0.26695						
*pseudo*F	3.6327	1.2491	8.6902	0.90222						
*R*^*2*^	0.32631	0.07242	0.53676	0.05338						
Pr (> F)	1e−04***	0.2038 ns	1e−04***	0.4289 ns						

	Bulk	Rhizosphere	CJ	PC	GN	Bulk	Rhizosphere	CJ	PC	GN
*d.f*	2	2	1	1	1	2	2	1	1	1
*SS*	1.469	0.966	0.351	0.398	0.225	1.578	1.593	0.407	0.243	0.103
*MS*	0.734	0.483	0.351	0.398	0.225	0.789	0.796	0.407	0.243	0.103
*pseudo*F	3.876	1.640	1.595	1.436	0.983	6.198	5.973	5.167	0.979	1.612
*R*^*2*^	0.564	0.354	0.285	0.264	0.197	0.674	0.666	0.564	0.197	0.287
Pr (> F)	0.004**	0.028*	0.1 ns	0.1 ns	0.5 ns	0.005**	0.003**	0.1 ns	0.6 ns	0.1 ns

MS, mean sum of squares; SS, sum of squares, significance value based on 999 permutations.

ns, no significance; *p-value < 0.05, **p-value < 0.01, *** *p-value* < 0.001; CJ, Cheongju; PC, Pyeongchang; GN, Gangneung.

**Figure 1:**
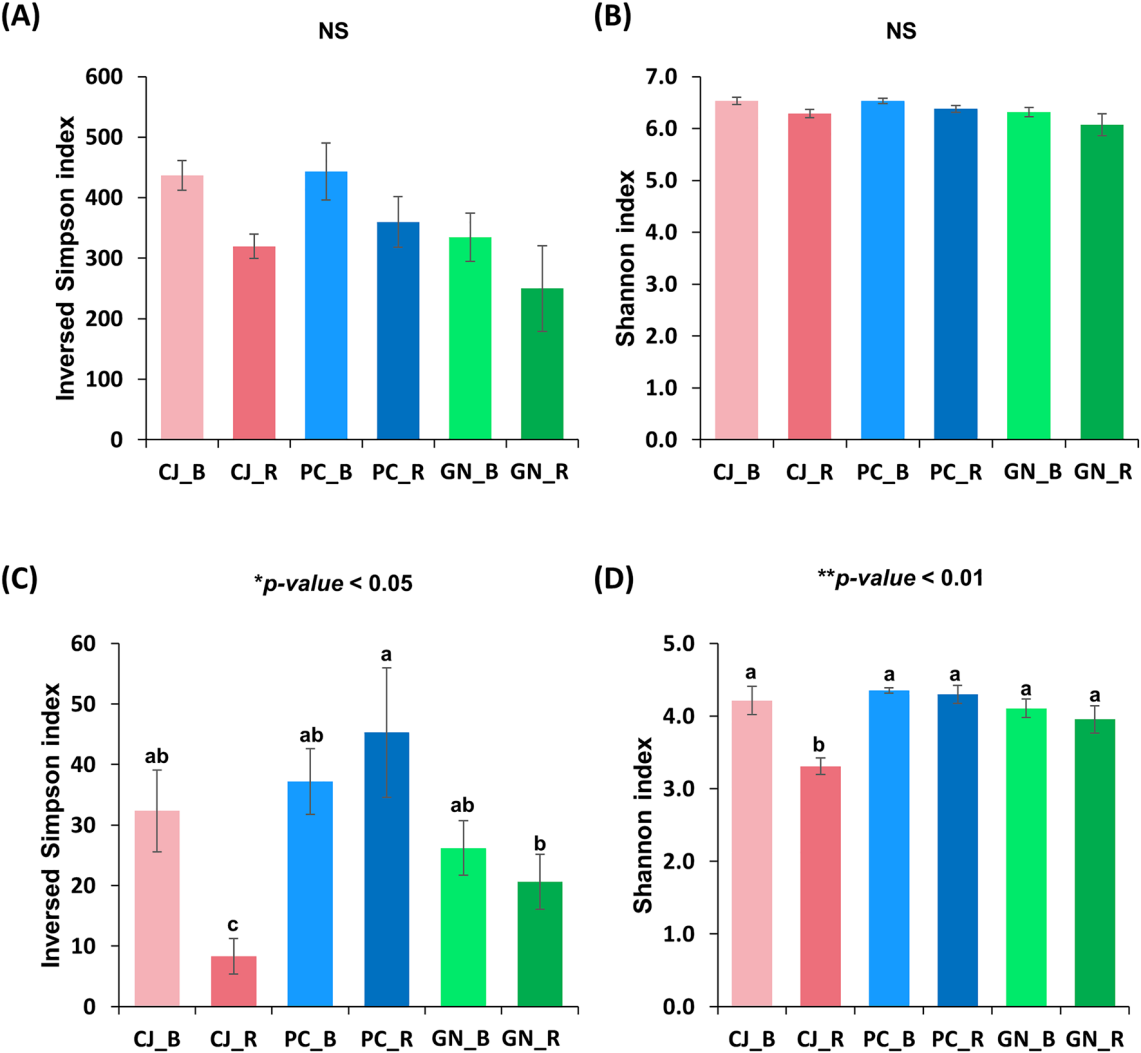
Simpson and Shannon indices of bacterial and fungal ASVs among three regions. (**A**) Bacterial inversed Simpson index. (**B**) Bacterial Shannon index. (**C**) Fungal inversed Simpson index. (**D**) Fungal Shannon index. CJ_B, Cheongju bulk; CJ_R, Cheongju rhizosphere; PC_B, Pyeongchang bulk; PC_R, Pyeongchang rhizosphere; GN_B, Gangneung bulk; GN_R, Gangneung rhizosphere. Error bar represent standard error. Statistical significance was determined using ANOVA. NS, no significance.

For the bacterial ASVs, over three times as many core (present in all three regional samples) rhizosphere ASVs (345) than in bulk soil samples (106). However a similar number of core fungal ASVs were detected in both bulk (71 ASVs) and rhizosphere soils (65 ASVs) (Supplemental Fig. S5). Bacterial and fungal diversities were analysed with Shannon and Simpson indices for each region (Fig. 1A–D). Bulk soil communities had a higher mean diversity compared to rhizosphere samples for a given region in both bacterial and fungal communities. The exception being fungal community diversity as interpreted by Simpsons index for Pyeongchang, which was higher in the rhizosphere samples (Fig. 1C). A previous potato microbiome study also reported lower bacterial diversity in rhizosphere soil while slightly higher diversity in the fungal community^25^. Hence, microbiota comparison between bulk and rhizosphere soils could be influenced by complex factors including soil type, climate, agronomy, sampling methods, and crop variety. More studies are therefore needed to identify the association between bulk and rhizosphere microbiota.

**Figure 2:**
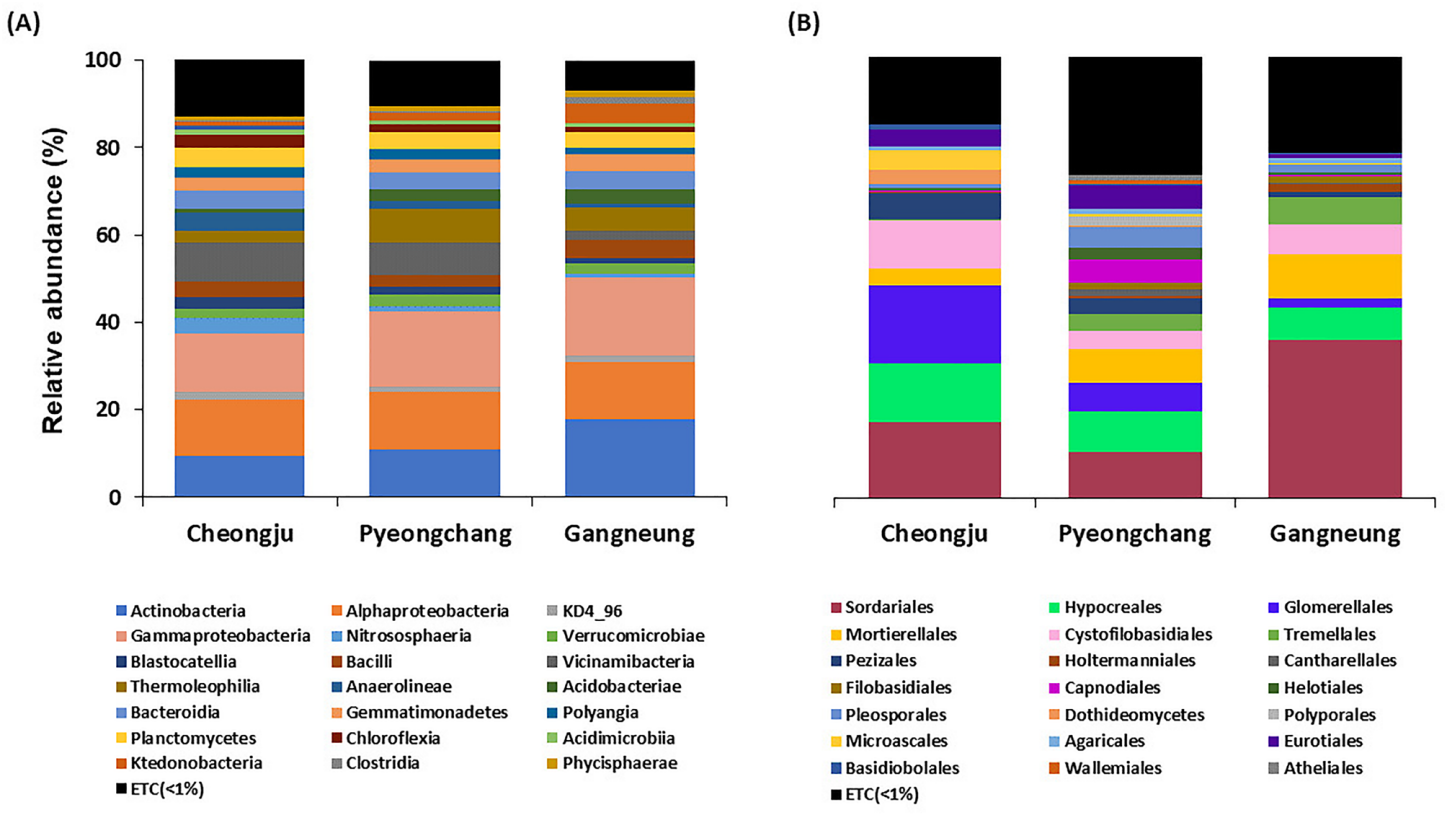
Relative abundance of three regions bacterial (**A**) and fungal (**B**) microbiotas. Bacterial and fungal ASVs are described in the class and order levels, respectively. ETC represents the sum of the relative abundances of ASVs that remain unidentified or constitute less than 1% of the total relative abundances.

Although there were no significant differences, Pyeongchang and Cheongju showed relatively higher bacterial diversity than Gangneung. For fungal diversity, Pyeongchang showed a significantly higher inversed Simpson index than other regions, though it showed the lowest observed number of ASVs detected (Supplemental Fig. S5, Supplemental Fig. S8) implying a reduced evenness due to the presence of dominant fungal population members. There are different calculation methods between Inversed Simpson and Shannon indices. Both methods consider abundance and evenness, but the Inversed Simpson index is more influenced by ASV proportion data. As such, ASVs with low abundance could exert less influence in Inversed Simpson index than in the Shannon index analysis. Hence, a significantly higher fungal inversed Simpson index in Pyeongchang with no significant difference in the Shannon index might be resulting from the exclusion of low abundant ASVs. This different in fungal structure may be directly related to the increase in yield observed in Pyeongchang and the exclusion of potential pathogen fungal species. The results of relative abundance also corresponded with that of (Fig. 2). Relative abundance described that more ASVs seem to be more equal in Pyeongchang compared to other regions, while some ASVs showed much higher relative abundances in both Cheongju and Gangneung in spite of higher unassigned and low abundant taxa of below 1% on Pyeongchang. The results of the Pielou’s evenness value described that significantly higher value in fungal ASVs of Pyeongchang than the other regions (Supplemental Table S2).

Relative abundance analysis was conducted on both bacterial and fungal communities in the class and order levels respectively (Fig. 2, Supplemental Fig. S9). The results of bacterial relative abundance showed higher abundances of Nitrososphaeria, Vicnamibacteria, Blastocatellia, and Anaerolineae while lower abundances of Gammaproteobacteria, Acidobacteria, and Thermoleophilia in Cheongju. For Pyeongchang, a higher abundance of Thermoleophilia and a lower abundance of KD4 96 were found. Gangneung showed higher abundances of Actinobacteria, Bacilli, and Ktedonobacteria while lower abundances of Blastocatellia and Vicinamibacteria.

Fungal relative abundance showed different results to bacterial relative abundance with higher diversity in Pyeongchang. For Cheongju, there were higher abundances of Hypocreales, Glomerellales, Cystofilobasidiales, Pezizales, and Microascales while Mortierellales showed lower abundance. For Pyeongchang, there were higher abundances of Capnodiales, Polyporales, Pezizales, Helotiales, Pleosporales, and Eurotiales and a lower abundance of Sordariales. Gangneung showed higher abundances of Sordariales, Mortierellales, and Tremellales while lower abundances of Hypocreales, Glomerellales, and Eurotiales. Arbuscular mycorrhizal (AM) fungi which are commonly observed in soil, have been shown to be common symbionts to plants, conferring many benefits in terms of nutrient uptake and tolerance to biotic or abiotic stress^26^. Within the fungal phyla Glomeromycota, many AM plant associations have been found and although they represent a very small proportion of fungal ASVs in this study, this group were most abundant in Pyeongchang soils (Supplemental Fig. S10).

### Potato yield and microbiome correlation analysis

**Figure 3:**
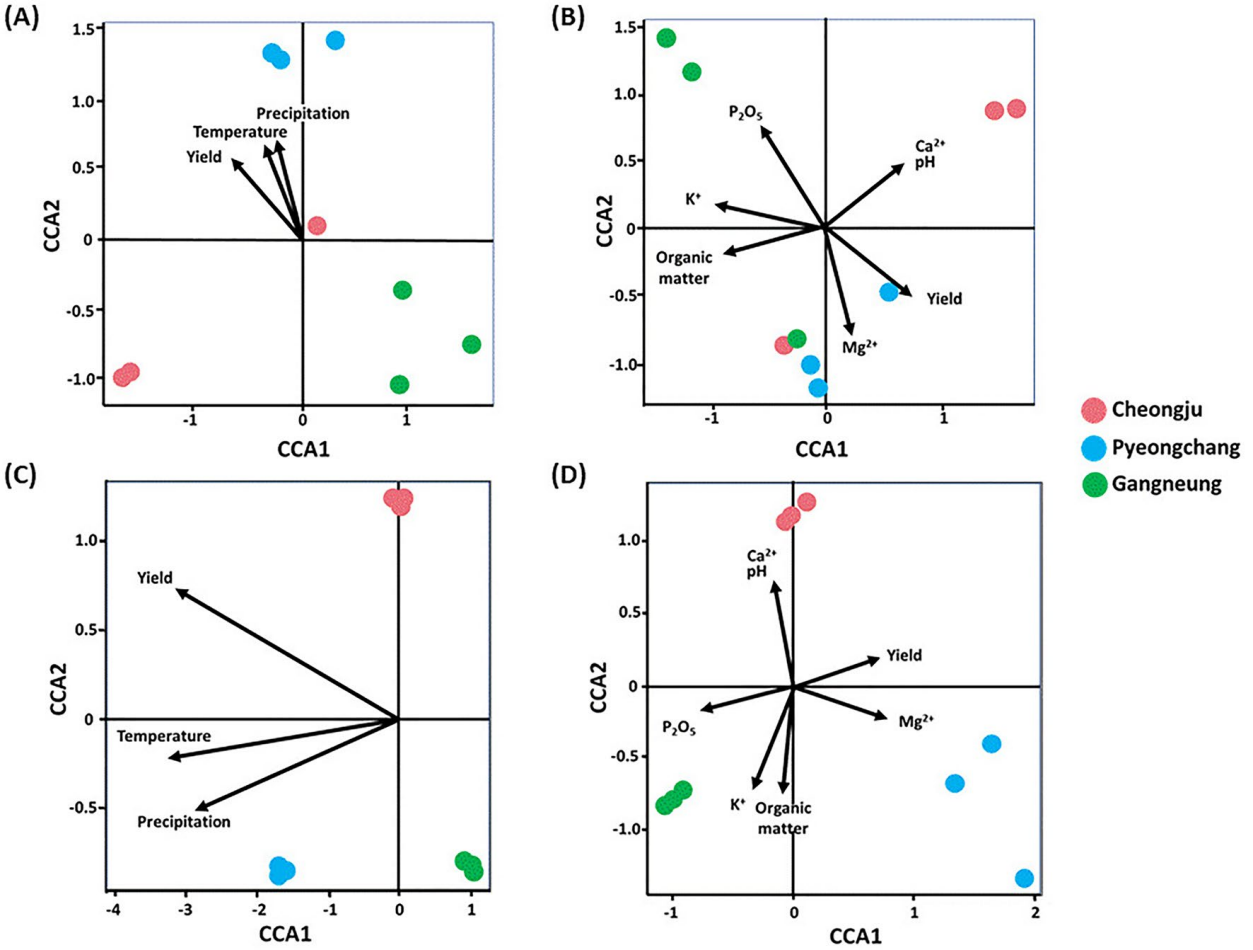
The results of canonical correspondence analysis on rhizosphere soil. (**A**) Bacterial ASVs, yield and climate conditions. (**B**) Bacterial ASV, yield and chemical properties. (**C**) Fungal ASVs, yield and climate conditions. (**D**) Fungal ASVs, yield and soil chemical properties. The distance between points reflects dissimilarities in microbiota. Each point represents each biological sample. Environmental variables are represented by arrows that point in the direction of increasing values of the variable.

One of the important goals in studying plant microbiota is their potential in stabilising crop production under different abiotic and biotic stresses^6^. In this research, we attempted to elucidate microbiota which are closely associated to potato yield by analysing how they and environmental factors relate to potato yield. The results of canonical correspondence analysis, which integrated environmental factors including average temperature, precipitation, soil characteristics, bacterial and fungal microbial groups and potato yield, revealed positive and negative correlation among some factors. The results described gradient influences of environmental factors on bacterial and fungal microbiota. Potato yield exhibited strong positive correlation with magnesium, precipitation, and temperature, with exceeding 0.8 of Spearman's correlation coefficients. These factors observed the highest values in Pyeongchang. In the other hand, soil-available phosphate which observed the highest value in Gangneung was negatively correlated with potato yield (Fig. 3, Supplemental Table S3).

Even though solar radiation was positively correlated to potato yield from previous work^18^, we found a stronger influence of temperature and precipitation on potato yield. Correlations among soil chemical factors were consistent with previous research results, showing a strong positive relation between pH and soil calcium, but except that it was inconsistent in soil chemical factors. Previous studies^27^ on the relationship between soil fertility and potato tuber nutrients have demonstrated the crucial role of soil pH, which showed a positive correlation with CEC (cation exchange capacity) involving elements such as potassium (K), calcium (Ca), and magnesium (Mg). On the other hand, the effects of nitrogen (N), phosphorus (P), and sulphur (S) on potato nutrients were found to be variable, influenced by factors like cropping systems and management practices. Comparing our findings and previous studies, we observed certain consistent results, particularly regarding the strong positive correlation between pH and calcium content. However, some of our results did not align with previous studies, which can be attributed to differences in the targeted soil. Previous studies collected samples from various soils, exhibiting a diverse range of pH levels and organic matter contents, whereas collected soil samples in our study were under optimum soil fertility for potato cultivation.

There was a negative correlation between yield and soil phosphate level. A previous study reported that phosphate efficiency for potato cropping is largely influenced by soil pH value, and different phosphate applications to potato cropping showed no significant difference in total tuber yield^28^. For soil magnesium a strong positive relationship to yield was observed as also previously reported by Marketa^29^. It suggests the possibility that higher soil magnesium could influence the higher microbial diversity in Pyeongchang. Thus, even though there are some positive and negative correlations between yield and soil chemical properties, other factors may be involved and a more cautious consideration is needed. The proportion of silt and clay also show strong positive relationships with yield describing 0.9 of correlation coefficients supported by Jeanne^30^ who reported that soil texture had a significant impact on potato yield (Supplemental Table S3).

**Figure 4:**
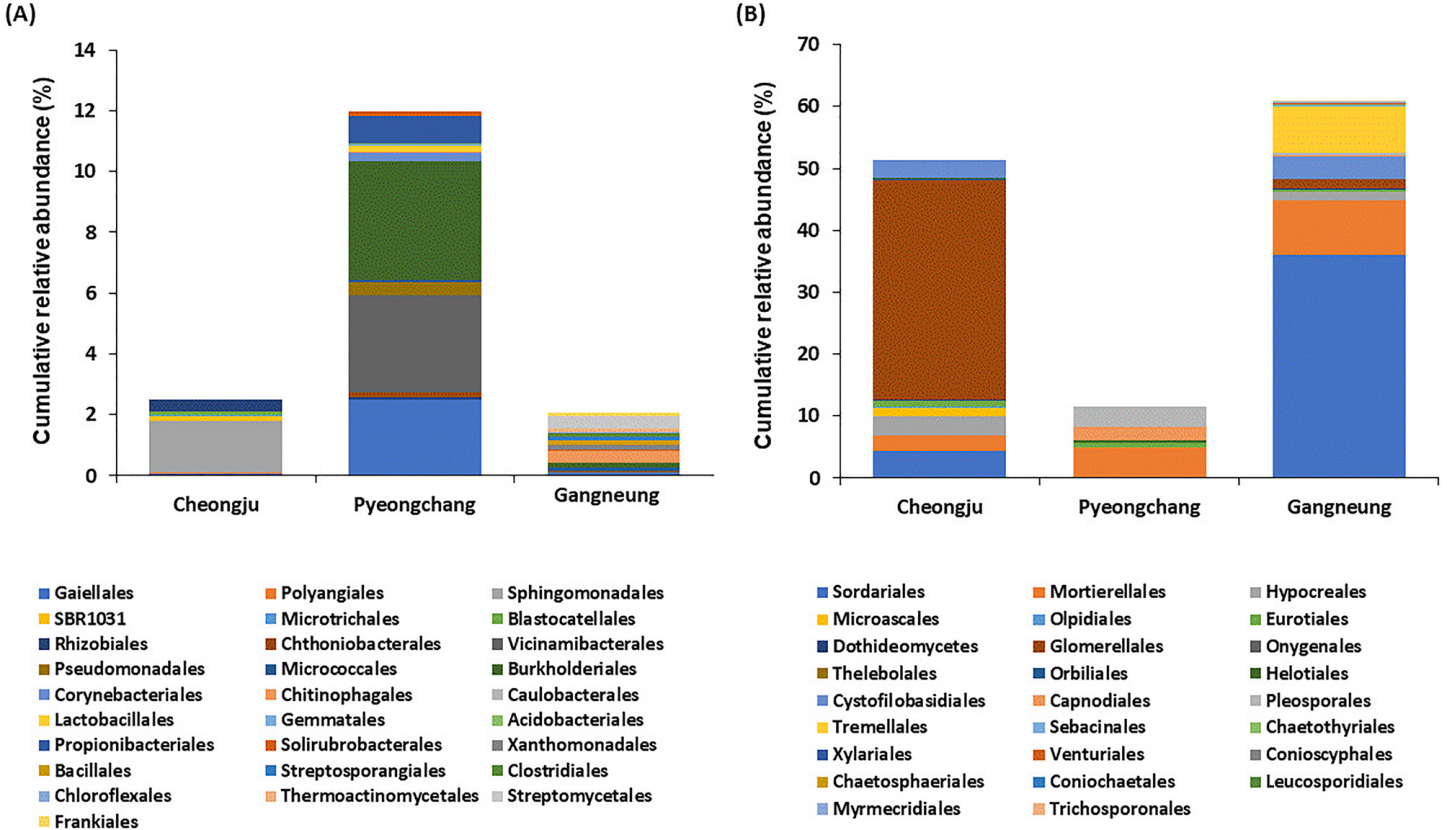
Cumulative indicator taxa abundance of bacterial (**A**) and fungal (**B**) communities on rhizosphere soil according to the three regions described in the order level.

Indicator analysis was also consistent with this result (Fig. 4). Indicator taxa analysis regarding three regions was conducted with the *indval* function in R program which calculates the indicator value based on relative abundance within clusters. The indicator ASVs with significant differences between regions were selected. Different regional ASVs detected as indicator taxa in bulk soil and rhizosphere soils showed few indicator ASVs were present in both bulk and rhizosphere soil. More indicator taxa were detected in bulk soil than rhizosphere soils for both bacterial and fungal ASVs (Supplemental Table S4). In Pyeongchang which showed the highest yields, fewer bacterial indicator ASVs were detected in the bulk soil, whilst the highest number of bacterial indicator ASVs were detected in rhizosphere soil. Meanwhile, there were the fewest number of fungal indicator ASVs in Pyeongchang in both bulk and rhizosphere soil. On the other hand, the largest number of indicator fungal ASVs were detected in Gangneung which showed the lowest yield. Although there was no significant differences of potato yields between Pyeongchang and Cheongju, no indicator ASVs were detected in across both regions. However, some bacterial and fungal genera were detected as indicator ASVs over all regions including *Sphingomonas*, *Nocardioides*, *Mortierella*, and *Penicillium*. In rhizosphere soil, the bacterial genera of *Sphingomonas* and *Nocardioides* was only detected as an indicator in Cheongju and Pyeongchang, respectively. Meanwhile, the fungal genera of *Mortierella* and *Penicillium* were detected in Cheongju and Pyeongchang, but not in Gangneung.

Among the indicator ASVs, all the ASVs positively related to yield were detected in Pyeongchang (Supplemental Table S4) while all the ASVs negatively related to yield were detected in Gangneung. Among 43 bacterial indicator ASVs identified in Pyeongchang, 30 ASVs were positively related to potato yield with over 0.7 of Spearman’s correlation coefficient. Throughout the bulk and rhizosphere soil samples, 8 and 2 ASVs of bacteria and fungi respectively were detected as yield-positive indicator ASVs in Pyeongchang (Supplemental Table S5). Although there were no bacterial indicator yield-negative ASVs detected from both bulk and rhizosphere soil in this region, many indicator ASVs of Gangneung showed a negative correlation with potato yield with 72 fungal yield-negative ASVs from both soil types in Gangneung (Supplemental Table S6).

Overall there were a smaller proportion of indicator ASVs from the bacterial community when compared to the fungal community. However, compared to the other regions, a higher proportion of bacterial indicator ASVs were detected from Pyeongchang consisting of Propionibacteriales, Burkholderiales, and Vicinamibacterales. Other bacterial indicator ASVs were among the Spingomonadales detected in Cheongju, while Chitinophagales and Streptomycetales were detected in Gangneung (Fig. 4A). Fungal indicator ASVs were proportionally far higher than bacteria in Gangneung. The higher proportion of Mortierellales and Pleosporales were identified as indicator taxa in Pyeongchang. Glomerellales showed remarkable proportions of indicator ASVs in Cheongju while higher proportions of Sordariales, Mortirellales, Cystofilobasidiales and Tremellales were detected in Gangneung (Fig. 4B).

Although the clusters defined at the class or phylum level are difficult to identify some functions related to plant health can be inferred. Many bacterial taxa belonging to Vicinamibacterales have been implicated as plant growth promoting bacteria involved in the nitrogen cycle^10,31^. Species belonging to Burkholderiales have been reported as plant growth promoting bacteria related to phosphorous cycle^32^. *Paraburkholderia*, *Cupriavidus* and *Massila* of Burkholderiales, which were detected in this research as putative yield-positive ASVs, have all been reported as plant growth promoting bacteria^33–35^, although *Massila* in this study showed a negative correlation with yield. *Rhizobium* and *Sphingomonas* are also well known genera with plant growth promoting functions^36^. Although there are few studies regarding Gaiellales, it is known that they support efficient soil nutrient cycling by degrading organic matter^37^.

Putative yield-negative fungal ASVs were associated with the lowest yield region which had reduced accumulated precipitation and drought stress. However, previous research has showed that no significant differences in bacterial and fungal rhizosphere microbiota observed under drought stress compared to control^38^, though other researchers have shown drought conditions affect soil microbiome either directly or indirectly^39,40^. More research is therefore required on rhizosphere microbiomes and their relationship to healthy crops, taking into account various factors, including not only climatic conditions but also crop effects such as, root exudates, varieties, plant developmental stages, and soil conditions. Only one paper so far^41^ has looked at potato microbiomes in relation to water stress. The putative yield-negative ASVs identified in this research should therefore consider whether they are potential plant pathogens, ASVs responding to drought conditions or have other detrimental impacts on plant health i.e. nutrient scavenging etc.

**Figure 5:**
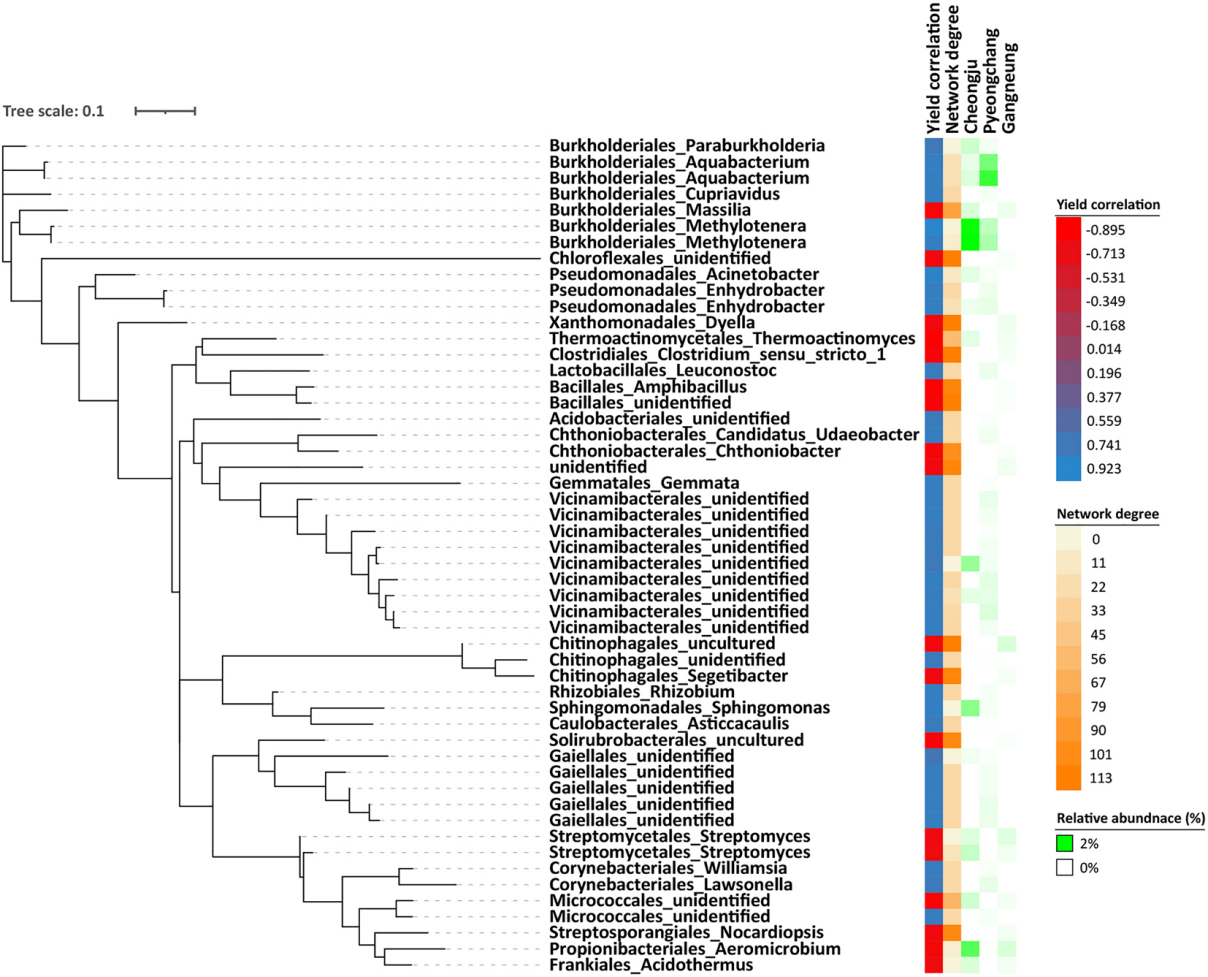
Phylogenetic tree regarding bacterial rhizosphere soil putative ASVs positively or negatively correlated to potato yield.

**Figure 6:**
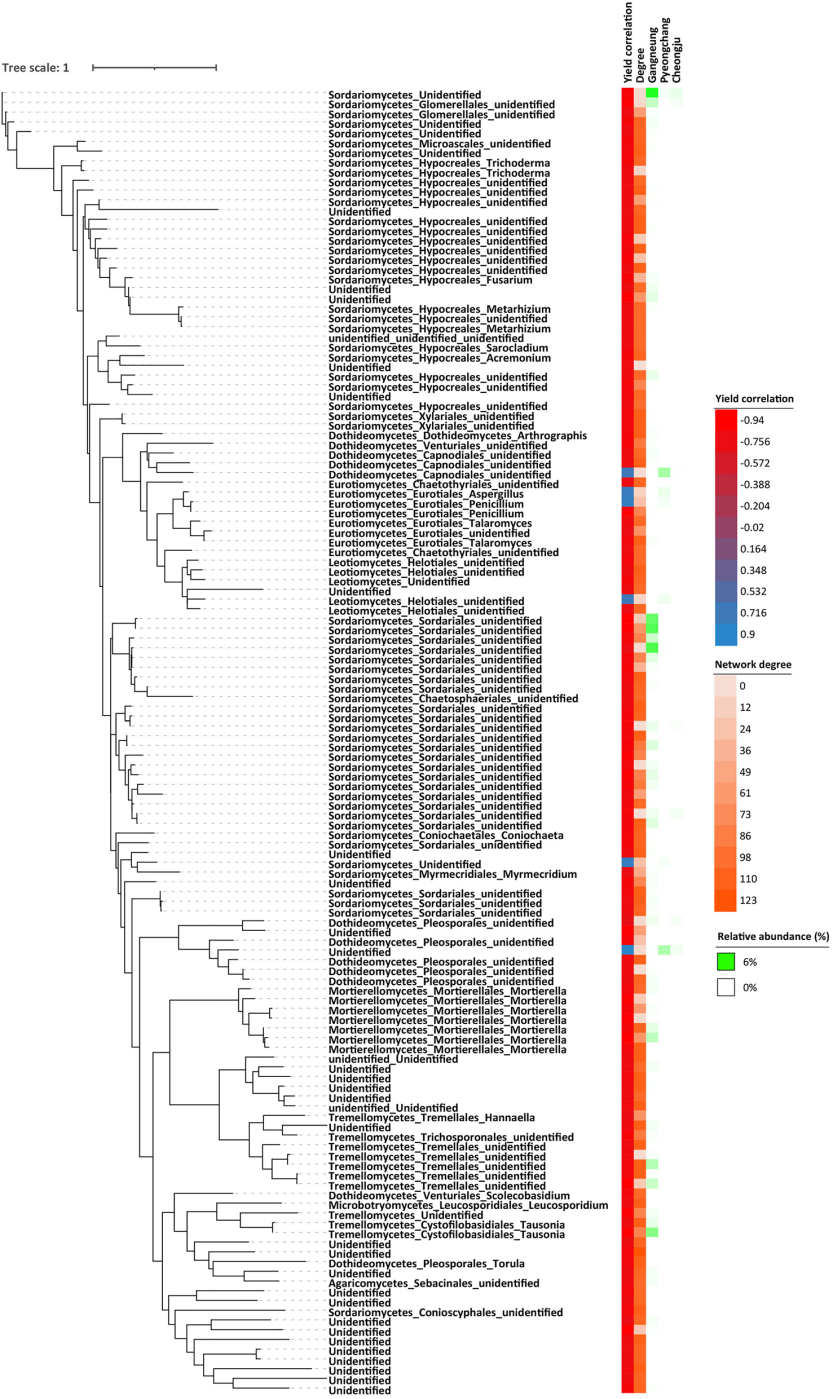
Phylogenetic tree regarding fungal rhizosphere soil putative ASVs positively or negatively correlated to potato yield.

Among 15,706 bacterial ASVs in potato rhizosphere soil, 34 ASVs were positively correlated to potato yield with over 0.7 correlation coefficient, while 19 ASVs were negatively correlated to potato yield below − 0.7 of a correlation coefficient. There were 4,089 fungal ASVs detected in the potato rhizosphere soil, with only 6 ASVs positively correlated to potato yield, while 128 ASVs were negatively correlated to potato yield (Supplemental Fig. S11). A greater number of bacterial ASVs are positively correlated with potato yield than fungal ASVs. In addition, the abundance of putative yield-positive ASVs showed a higher correlation with Pyeongchang which also had the highest potato yield, while the abundance of putative yield-negative ASVs showed a greatest correlation with Gangneung which also generated the lowest potato yield (Figs. 5 and 6). Therefore, the results were consistent with indicator analysis. Most putative yield-negative fungal ASVs were however unable to be identified at the genus level. Within the microbial network, putative yield-negative ASVs exhibited a higher degree of node compared to putative yield-positive ASVs, implying a more intricate relationship within the potato rhizosphere soil microbiome.

Although many fungal ASVs were unable to be identified, the plant pathogen *Fusarium* was identified as well as *Penicillium* which has been reported as both a plant pathogen and to have plant growth promoting functions^42,43^. The putative yield-negative fungus with the highest number of ASVs was Sordariales, and increased in response to drought stress, as did *Streptomyces*, previously detected under drought conditions^44,45^. Some known plant pathogens from the orders Pleosporales and Hypocreales^46,47^, were detected as putative yield-negative ASVs. Additional *Mortierella* associated putative yield-negative ASVs were detected, however these have previously been were reported as plant growth-promoting fungi in agricultural soils^48^.

### Core ASVs analysis of potato rhizosphere soil

**Figure 7:**
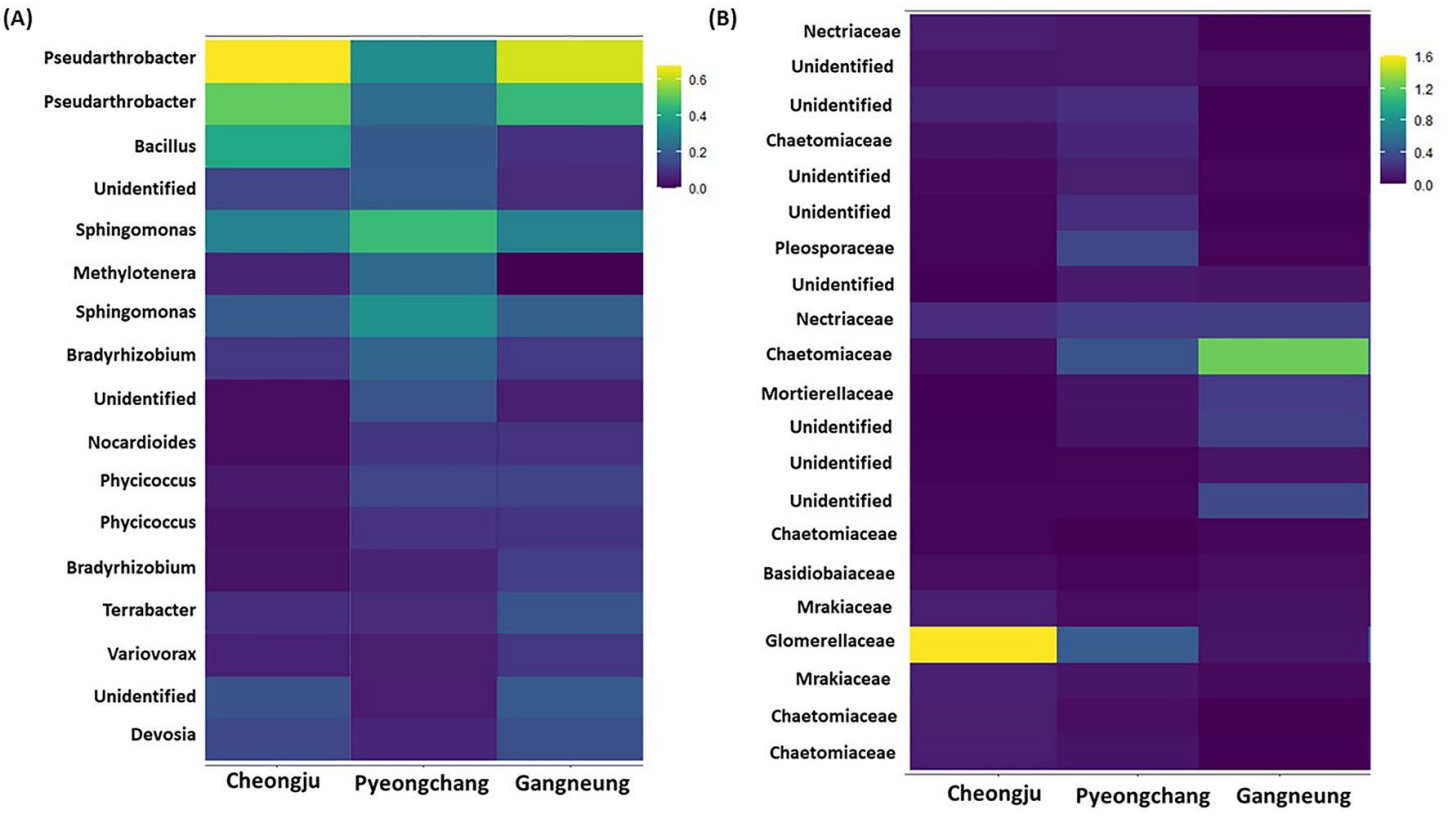
Core bacterial (**A**) and fungal (**B**) ASVs heatmap of potato rhizosphere soil over three regions. Colour bar represents abundances of ASVs.

The indicator ASVs described so far are either positively or negatively correlated to yield or other regional or environmental factors across the three regions, they do not therefore necessarily represent the core rhizosphere potato ASVs. The core ASVs have been used widely in microbiome research with various meaning, but generally, the core ASVs indicate the ASVs associated with the host^49^. Hence, we suggested 17 bacterial ASVs and 21 fungal ASVs as the potato rhizosphere core ASVs in spite of different environmental conditions (Fig. 7).

Even if some ASVs are not identified yet, *Pseudarthrobacter*, *Bacillus, Sphingomonas, Methylotenera, Bradyrhizobium, Nocardioides, Phycicoccus, Terrabacter, Vriovorax* and *Devosia* were detected as core bacterial taxa on rhizosphere soil of potato cv. Sumi. On the other hand, *Nectriaceae, Chaetomiaceae, Pleosporaceae, Mortierellaceae, Basidiobaiaceae*, *Glomerellaceae* and *Mrakiaceae* are described as the core fungal taxa. Although few studies have researched the potato microbiota, potato bacterial and fungal microbiota were clearly distributed depending on the regions, cultivars, and soil types^9,16,41,50^. Because they did fungal core taxa at the class level, it is difficult to compare to this current research. However, *Bradyrhizobium, Sphingomonas,* and *Devosia,* which were detected as core bacterial taxa in this research work, were also described as core bacterial taxa in previous research.

This work has highlighted key bacterial and fungal taxa that are potentially associated with high and low yields of potato in relation to metadata which includes, altitude, soil chemical and physical parameters as well as weather data. Taken together this information can be used to test the field environment prior to as well as during cultivation of this crop in order to guide what agronomic practices should be used to ensure successful agricultural production and to assess whether potatoes are a suitable crop for a given environment. The use of core microbial taxa that show increased or decreased abundance in response to crop yield could be considered as putative target organisms to test system yield potential. Based on the comprehensive analysis of this study, we tentatively propose the possibility that *Rhizobium* and *Sphingomonas* may be associated with potato productivity or aspects related to potato productivity in the environment. However, it is important to acknowledge that environmental factors such as soil physiochemical properties and soil moisture contents are crucial influencers of potato yield and soil microbiota. Therefore, additional experiment are needed to identify the direct positive roles of* Rhizobium* and *Sphingomonas* groups in enhancing potato yield.

## Materials and methods

### Potato cultivation in three different regions of Korea

To analyse the relationship between soil microbiota, environmental conditions, and potato growth and yield, potato fields in three different climatic regions of Korea were selected: Cheongju, Pyeongchang, and Gangneung. Cheongju (36.72644559264173, 127.46206046548588) is located in the middle of Korea and potatoes are cultivated during spring from the middle of March to the end of June. Pyeongchang (37.682555689366545, 128.7282038575868) is located in a mountainous area, 700 m altitude, and cultivated from the middle of May to the end of August. Gangneung (37.776176861264474, 128.93208462616758) is located near the East Sea of Korea and potato cultivation is from the end of March to the end of June. The same cultivar and field management were applied to each of the three regions. Two weeks before planting the seed potatoes, compound chemical fertilizer (Nitrogen-Phosphorous-Potassium at 21, 17, and 17%, 500 kg/ha) and compost (20,000 kg/ha) were added to the potato field and rotary tillage was applied. For potato cultivation, the variety Sumi produced by the Highland Agriculture Research Institute was used. Seed potatoes (variety Sumi, supplied by Highland Agriculture Research Institute) were planted at 80 × 25 cm apart distances with black vinyl mulching.

### The data of Weather and soil moisture contents collection

Precipitation, solar radiation, and temperature data were collected by the meteorological administration of Korea (https://data.kma.go.kr) and altitude verified with google earth (https://earth.google.com). The average conditions over 20 years were calculated from 1996 to 2015. As the potato cultivation period of each region is slightly different, Cheongju and Gangneung between March to June while Pyeongchang between May to August, the precipitation and solar radiation values were aggregated while average values were applied for temperature during the potato cultivation period for each region (Supplemental Table S1).

The soil moisture contents data were collected by the Rural Development Administration of Korea and courced from https://weather.rda.go.kr. The locations were selected to be as close as possible to each experimental field: Cheongju (code: 365803A001, 36.85385, 127.42977), Gangneung (code: 240813A001, 37.4852, 129.11882) and Pyeongchang (code: 235802A001, 37.20634, 128.9857). Notably, the selected location of Pyeongchang is at an altitude of 700 m, which is similar to the altitude of experimental field in Pyeongchang. Based on potato cultivation periods, soil moisture data were collected from end of March to end of June for spring potato in Cheongju and Gangneung. For Pyeongchang, where summer potato was cultivated, the soil moisture data were collected from mid-May to the end of August. The average soil moisture contents from the flowering stage to harvest were presented in Supplemental Fig. S3. Specifically, the data from 15 May to 30 June were used for spring potato, whereas the data from 15 July to 31 August were used for summer potato.

### Soil and rhizosphere sampling

Soils for chemical and textural analysis were collected prior to fertilization and rotary. One experimental field was selected for each region. Five core soil samples were randomly collected with an auger (0–15 cm of depth) for each field and placed into sampling bags. Collected soil samples were mixed and pooled on a tray before air dried for 48 h in the shade. They were then sieved at 2 mm and homogenized before soil chemical and texture analysis.

For microbiome analysis, two different niches, bulk soil and rhizosphere soils were collected at flowering stage. All tools used in the sampling were sterilized with 70% ethanol before sampling. Bulk soils were collected to a depth of 5–20 cm between potato rows using a spade. Five samples of bulk soil were collected into the same bag, and thoroughly mixed. The mixed bulk soil was transferred into 15 ml of sterilized falcon tube for storage. For rhizosphere soil sampling, the potato roots were cut with sterilised scissors and vigorously shaken using a pair of tweezers to remove loosely adhering soil. The root-adhering rhizosphere soil was submerged in 15 ml of sterile water in a 50 ml Falcon tube. Tubes were shaken for 30 s by hand. The soil suspension was centrifuged at 5000 × g for 5 min and supernatant discarded. In this research, microtiota analysis was conducted on three rhizosphere soils and three bulk soils. For bulk soil analysis, DNA extraction was performed three times using homogenized bulk soil collection. Regarding rhizosphere soil analysis, one rhizosphere soil sample was collected from the root of each potato plant. Collected bulk soil and rhizosphere soil were stored at – 70 °C prior to DNA extraction.

### Soil analysis for chemical properties and texture

To elucidate soil chemical properties, soil pH, amount of organic matter, available phosphate, exchangeable potassium, calcium, and magnesium were determined. Soil pH was measured using an electrometric method in a 1:5 (w/v) of soil:water ratio with a pH meter (Orion Versa Star Pro, Thermo Scientific, Massachusetts, USA). Soil organic carbon was measured with a C/N analyzer (Vario Max Cube Elementar, Germany). Available phosphate was determined by the Lancaster method^51^ using a UV/VIS spectrometer (Lamda 25, Perkinelmer Co., Norwalk, CT. USA). Exchangeable cations were measured by inductively coupled plasma spectrometry (Optima 2100DV, PerkinElmer Co., Norwalk, CT, USA) using 1 M of NH_4_OAc solution soil extract. Soil textures were determined by the Hydrometer method^52^. Soil texture classification for each region was based on the soil texture triangle classified by USDA^53^.

### Yield investigation

The investigation of potato yield was conducted at the harvest stage, three months after planting. In each experimental field area, three blocks were set up and managed. Ten plants were sampled from each blocks to measure the weight of potato tubers. Based on the investigated data, the yield data was calculated per hectacres (equivalent to 10,000 m^2^), taking into account the planting distances of 80 × 25 cm. The average value and standard error of three blocks were presented as Supplemental Fig. S1.

### Soil microbiota analysis

The DNA from bulk soil or rhizosphere soil was extracted using the ISOIL for beads beating DNA extraction kit (Nippongene, Japan) following the manufacturer’s instructions. Extracted DNA was stored at – 20 °C prior to PCR amplification. For bacterial microbial community profiling, the V4 region of the 16S rRNA gene was amplified using the universal primers: 515F^54^ (5’-TCGTCGGCAGCGTCAGATGTGTATAAGAGACAG-GTGCCAGCMGCCGCGGTAA-3’) and 806R^55^ (5’-GTCTCGTGGGCTCGGAGATGTGTATAAGAGACAG-GGACTACHVGGGTWTCTAAT-3’). For fungal community structure analysis, universal primer pairs for ITS1 spacer region were used: ITS1F (5’-TCGTCGGCAGCGTCAGATGTGTATAAGAGACAG-CTTGGTCATTTAGAGGAAGTAA-3’) and ITS2R (5’-GTCTCGTGGGCTCGGAGATGTGTATA AGAGACAG-GCTGCGTTCTTCATCGATGC-3’) for ITS1 amplification^56^.

PCRs were performed using AmpliTaq GOLD (Applied Biosystems; Carlsbad, CA, USA) and PCR conditions were as follows: (1) 95 °C for 10 min, (2) 95 °C for 30 s, (3) 55 °C for 30  s, (4) 72 °C for 1 min and (5) 72 °C for 7 min, repeated (2)–(4) 30 cycles. Illumina sequencing library preparation was performed as per the Illumina, 2013, Illumina Co., California, USA, using Nextera barcodes. Prepared libraries were sequenced by Macrogen Co. (Seoul, Korea) using the MiSeq platform (Illumina Co., California, USA). Sequence data was returned as demultiplexed fastq paired-end files.

### Data analysis

Microbial sequence data were analysed using the QIIME2 platform for merging, denoising, and taxonomic assignment to Amplicon Sequence Variants (ASV) with SILVA 138 for bacteria and UNITE v8 databases for fungi. Analyses of the microbiota for α-diversity (Shannon, Simpson, observed ASVs), β-diversity (unweighted and weighted UniFrac, PCoA), permutational multivariate analysis of variance (PERMANOVA), canonical correspondence analysis, indicator taxa analysis, and core taxa analysis were conducted with phyloseq, microbiome, vegan and labdsv packages in R (4.1.1). Relative abundance analysis was calculated and described using Excel and Venn diagrams generated using Venny 2.1. All correlation values were measured with R using Spearman’s correlation. From the results, the data which showed over 0.7 of the absolute value of Spearman’s correlation coefficient were selected for further analysis. Network analysis was conducted with rhizosphere ASVs using Hmisc package in R. Phylogenetic trees for both bacteria and fungi were constructed in iTOL (https://itol.embl.de) using rhizosphere ASVs.

### Ethical approval

The research conducted adhered to the guidelines outlined in the IUCN Policy Statement on Research Involving Species at Risk of Extinction and Convention on the Trade in Endangered Species of Wild Fauna and Flora.

### Supplementary Information

Supplementary Information.

## Data Availability

The datasets generated and/or analysed during the current study are available in the KNB (The Knowledge Network for Biocomplexity) repository. Access link: https://knb.ecoinformatics.org/view/urn%3Auuid%3A7e5e3b7d-83c1-41ac-940c-414ef414faa5.
