# Supplementary material for: The potato rhizosphere microbiota correlated to the yield of three different regions in Korea
Source: Sci Rep. 2024 Feb 24;14:4536. doi: 10.1038/s41598-024-55263-7 (PMC10894198; doi:10.1038/s41598-024-55263-7)
Supplement: Supplementary file 1 — Supplementary Information. [file 41598_2024_55263_MOESM1_ESM.pdf]

Supplemental Table S1. The weather conditions among three regions during potato cultivation

Region	Year	Temperature (°C)	Accumulated precipitation (mm)	Cumulative solar radiation (MJ/m^2)	Altitude (m)
Cheongju	2016	16.3	269.6	2186.4	52
	20 years average	15.3	380.9	2054.5	
Pyeongchang	2016	17.4	545.0	2229.6	766
	20 years average	17.1	954.7	1942.7	
Gangneung	2016	15.8	176.6	2305.5	7
	20 years average	14.8	358.1	1998.1	

Supplemental Table S2. The average value and statistical analysis (ANOVA) results of the Pielou's evenness of bacterial and fungal ASVs among three regions

	Cheongju	Pyeongchang	Gangneung	*F-value*	*p-value*
Bacteria	0.9475[a]	0.9489[a]	0.9376[a]	1.8213	0.1958 ns
Fungi	0.6392[b]	0.8641[a]	0.6830[b]	28.5109	7.76e-06***

ns: no significance, *p-value* < 0.001

Supplemental Table S3. The correlation analysis between potato yield and environmental conditions. Spearman correlation coefficients

	Spearman's rho	*p-value*		Spearman's rho	*p-value*
Temperature	0.95	9.59E-05	Soil pH	-0.44	0.2395
Precipitation	0.95	9.59E-05	Soil phosphate	-0.87	0.0024
Solar radiation	-0.47	0.197	Soil organic matter	0	1
Clay	0.95	9.59E-05	Soil K	-0.48	0.1942
Silt	0.95	9.59E-05	Soil Mg	0.88	0.0015
Sand	-0.47	0.197	Soil Ca	-0.48	0.1875

Supplemental Table S4. The number of indicator ASVs on bulk soil and rhizosphere soil according to the regions

Kingdom	Niche	Cheongju	Pyeongchang	Gangneung	Total
Bacteria	Bulk soil	298(0/0)	31(30/0)	166(0/120)	495
	Rhizosphere soil	10(0/0)	43(30/0)	19(0/17)	72
Fungi	Bulk soil	117(0/0)	18(10/0)	148(0/144)	283
	Rhizosphere soil	58(0/0)	10(7/0)	119(0/115)	187

* The numbers in the bracket represent the number of yield-positive ASVs and yield-negative ASVs.

Supplemental Table S5. Yield-positive of Pyeongchang indicator ASVs throughout the bulk and rhizosphere soil. R^2 is spearman's correlation coefficients with yield

Kingdom	No	R^2	Phylum	Class	Order	Family	Genus
Bacteria	1	0.84	Proteobacteria	Gammaproteobacteria	Burkholderiales	Comamonadaceae	Aquabacterium
	2	0.84	Proteobacteria	Gammaproteobacteria	Burkholderiales	Comamonadaceae	Aquabacterium
	3	0.84	Proteobacteria	Gammaproteobacteria	Burkholderiales	Methylophilaceae	Methylotenera
	4	0.92	Proteobacteria	Gammaproteobacteria	Burkholderiales	Methylophilaceae	Methylotenera
	5	0.84	Proteobacteria	Gammaproteobacteria	Pseudomonadales	Moraxellaceae	Enhydrobacter
	6	0.82	Proteobacteria	Gammaproteobacteria	Pseudomonadales	Moraxellaceae	Enhydrobacter
	7	0.78	Bacteroidota	Bacteroidia	Chitinophagales	Chitinophagaceae	unidentified
	8	0.78	Proteobacteria	Alphaproteobacteria	Caulobacterales	Caulobacteraceae	Asticcacaulis
Fungi	1	0.82	Ascomycota	Sordariomycetes	unidentified	unidentified	unidentified
	2	0.82	Ascomycota	Eurotiomycetes	Eurotiales	Aspergillaceae	Penicillium

Supplemental Table S6. Yield-negative of Gangneung indicator ASVs throughout the bulk and rhizosphere soil (Top 15 rank by the abundance of 77 numbers of fungal ASVs). R^2 is spearman's correlation coefficients with yield

Kingdom	No	R^2	Phylum	Class	Order	Family	Genus
Bacteria	1	-0.74	Actinobacteriota	Actinobacteria	Frankiales	Acidothermaceae	unidentified
	2	-0.76	Chloroflexi	KD4_96	unidentified	unidentified	unidentified
Fungi	1	-0.48	Ascomycota	Sordariomycetes	Sordariales	Chaetomiaceae	unidentified
	2	-0.5	Mortierellomycota	Mortierellomycetes	Mortierellales	Mortierellaceae	Mortierella
	3	-0.89	Basidiomycota	Tremellomycetes	Cystofilobasidiales	Mrakiaceae	Tausonia
	4	-0.77	Ascomycota	Sordariomycetes	Sordariales	Lasiosphaeriaceae	unidentified
	5	-0.59	Basidiomycota	Tremellomycetes	Tremellales	unidentified	unidentified
	6	-0.91	Ascomycota	Sordariomycetes	Sordariales	Lasiosphaeriaceae	unidentified
	7	-0.69	Mortierellomycota	Mortierellomycetes	Mortierellales	Mortierellaceae	Mortierella
	8	-0.91	Mortierellomycota	Mortierellomycetes	Mortierellales	Mortierellaceae	Mortierella
	9	-0.89	Ascomycota	Sordariomycetes	Glomerellales	Plectosphaerellaceae	unidentified
	10	-0.84	Ascomycota	Sordariomycetes	Sordariales	Lasiosphaeriaceae	unidentified
	11	-0.85	Ascomycota	Sordariomycetes	Sordariales	Chaetomiaceae	unidentified
	12	-0.82	Ascomycota	Sordariomycetes	Sordariales	Chaetomiaceae	unidentified
	13	-0.84	Mortierellomycota	Mortierellomycetes	Mortierellales	Mortierellaceae	Mortierella
	14	-0.91	unidentified	unidentified	unidentified	unidentified	unidentified
	15	-0.78	Basidiomycota	Tremellomycetes	Cystofilobasidiales	Mrakiaceae	Tausonia

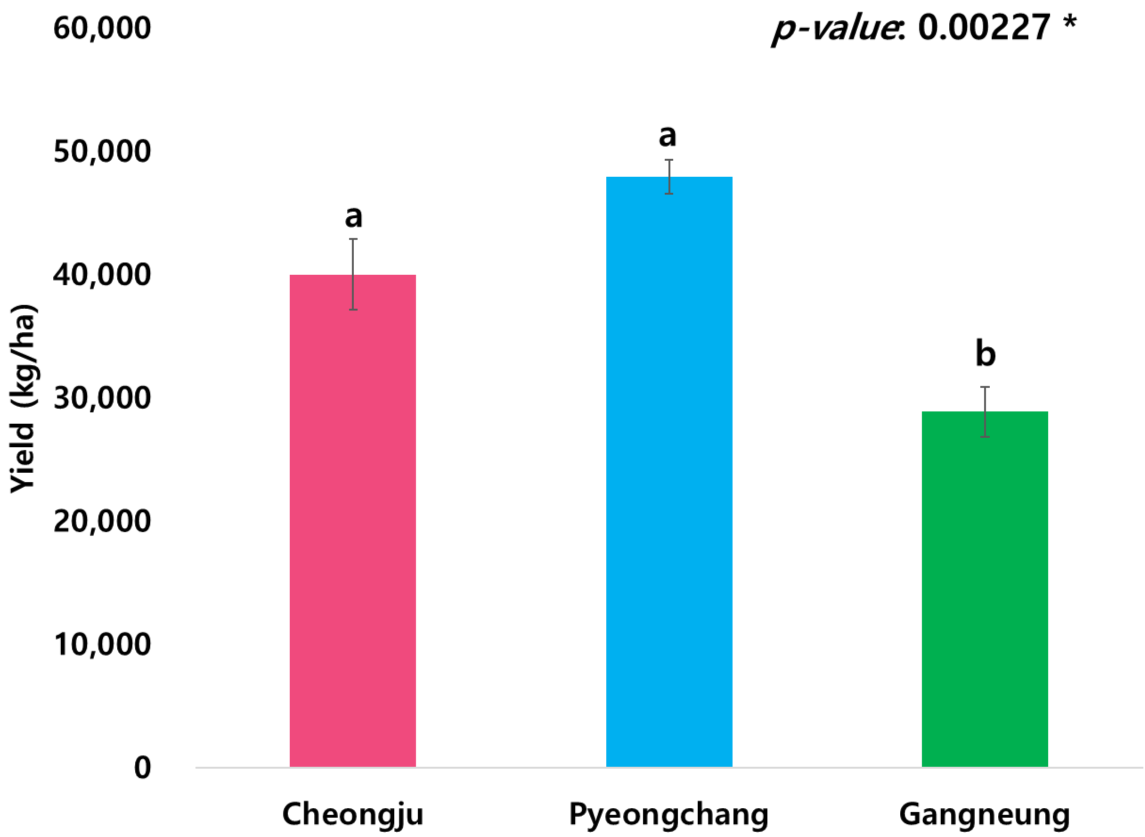

Supplemental Figure S1. Potato yield of three regions in 2016, Error bar represent standard error, ANOVA

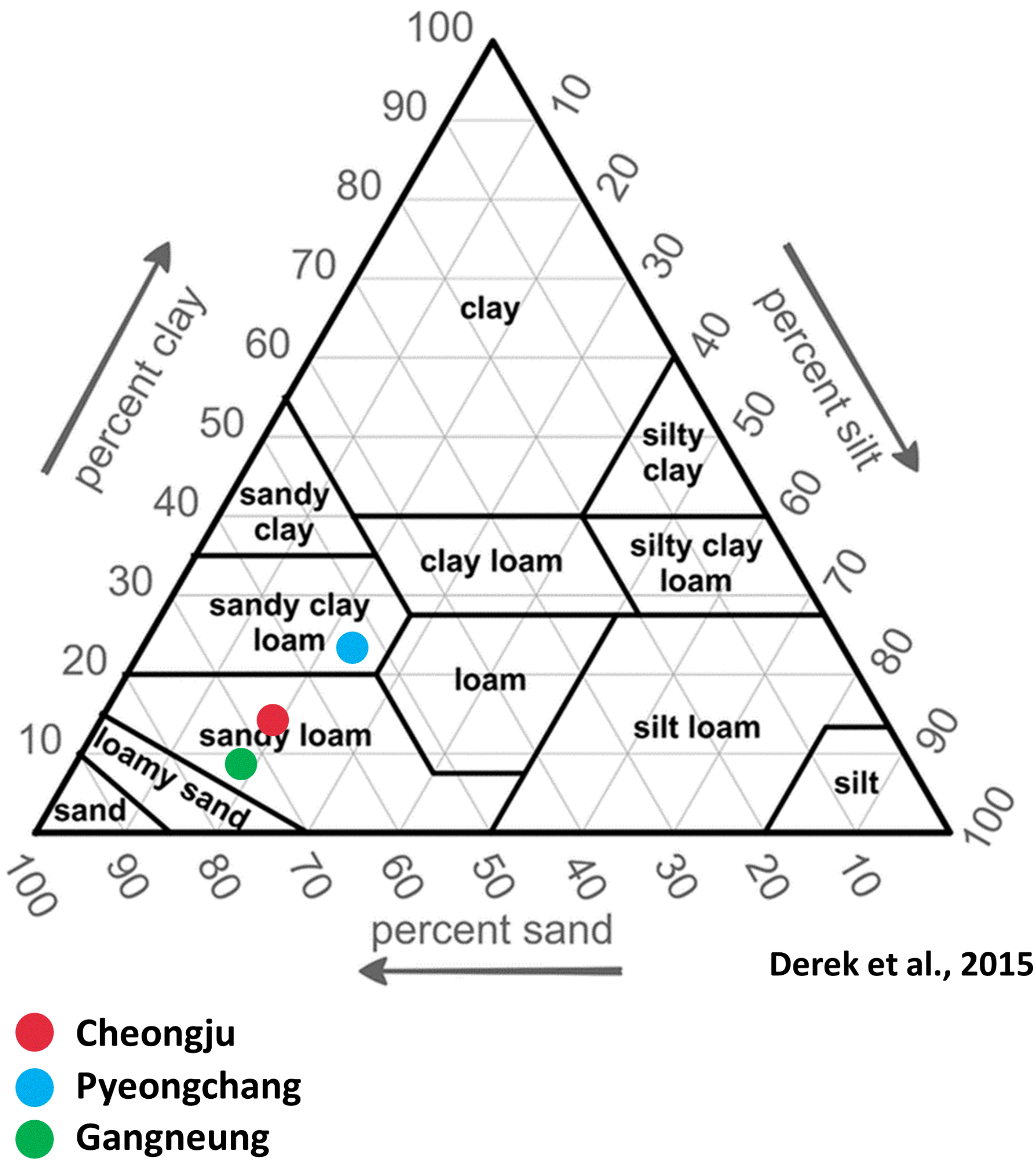

Supplemental Figure S2. The proportions of clay, silt and sand among the three regions

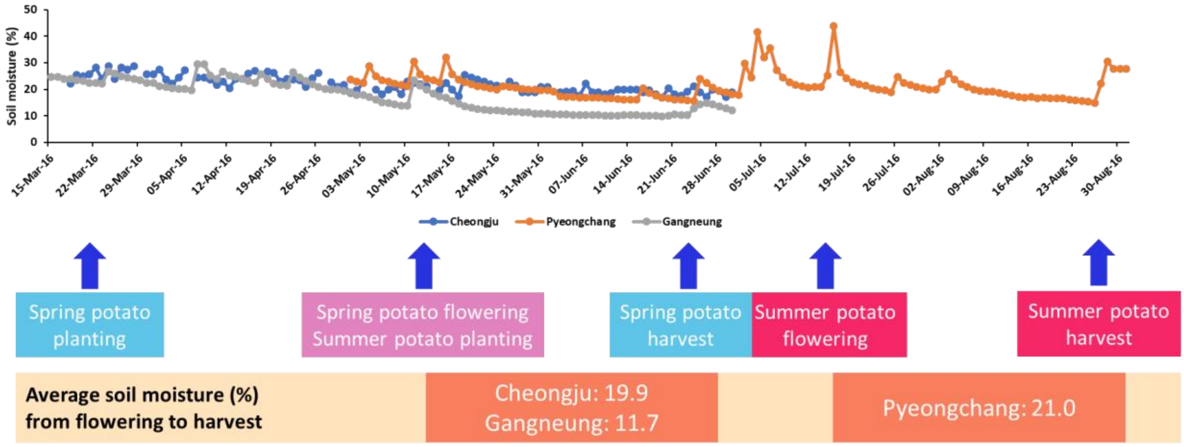

Supplemental Figure S3. The soil moisture contents during potato cultivation periods on three regions

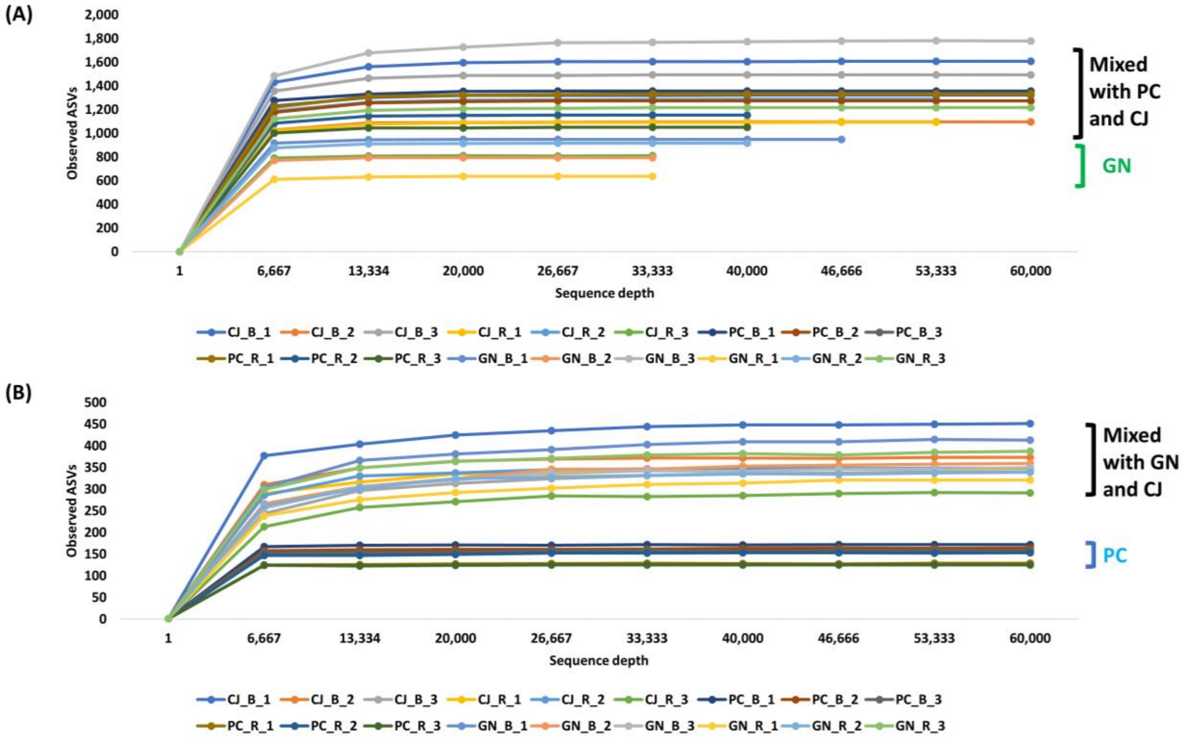

Supplemental Figure S4. Rarefaction curve of bacterial (A) and fungal (B) observed ASVs, CJ_B: Cheongju bulk, CJ_R: Cheongju rhizosphere, PC_B: Pyeongchang bulk, PC_R: Pyeongchang rhizosphere, GN_B: Gangneung bulk, GN_R: Gangneung rhizosphere

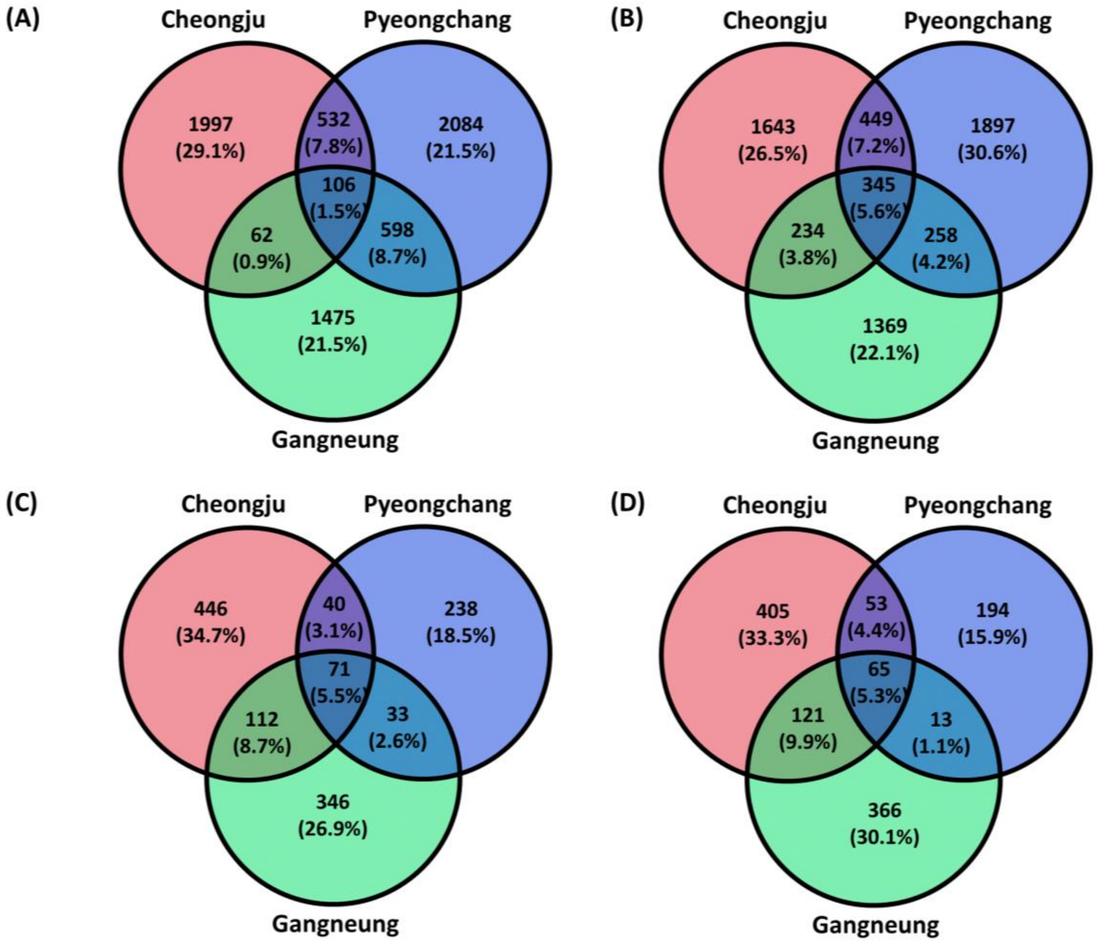

Supplemental Figure S5. The numbers of observed bacterial bulk soil (A), bacterial rhizosphere soil (B), fungal bulk soil (C) and fungal rhizosphere soil (D) ASVs according to regions (Venn diagram analysis)

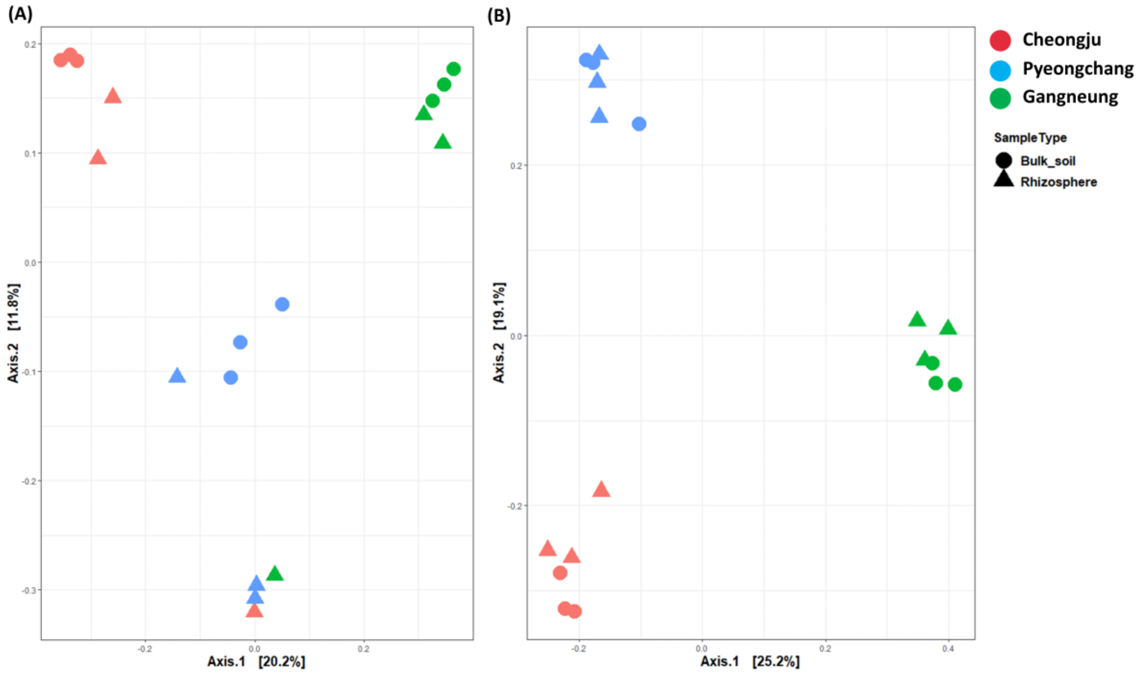

Supplemental Figure S6. β-diversity of bacterial (A) and fungal (B) microbiota according to regions and niches. β-diversity of bacterial and fungal communities were determined using an unweighted UniFrac distance-based principal coordinate analysis. Dots indicate replicates of each location and niche. The shapes of dots represent different niches (filled triangle, rhizosphere; filled circle, bulk soil). Colours of dots correspond to each geographic location (red, Cheongju; blue, Pyeongchang; green, Gangneung)

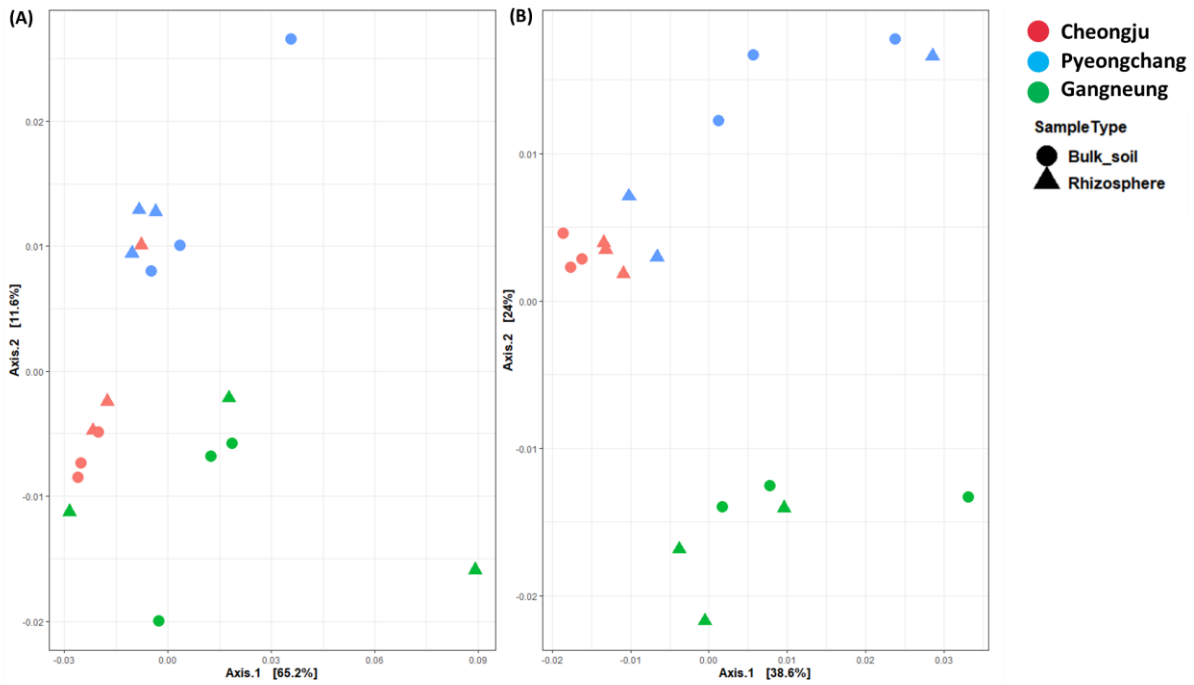

Supplemental Figure S7. β-diversity of bacterial (A) and fungal (B) microbiota according to regions and niches. β-diversity of bacterial and fungal communities were determined using an weighted UniFrac distance-based principal coordinate analysis. Dots indicate replicates of each location and niche. The shapes of dots represent different niches (filled triangle, rhizosphere; filled circle, bulk soil). Colours of dots correspond to each geographic location (red, Cheongju; blue, Pyeongchang; green, Gangneung)

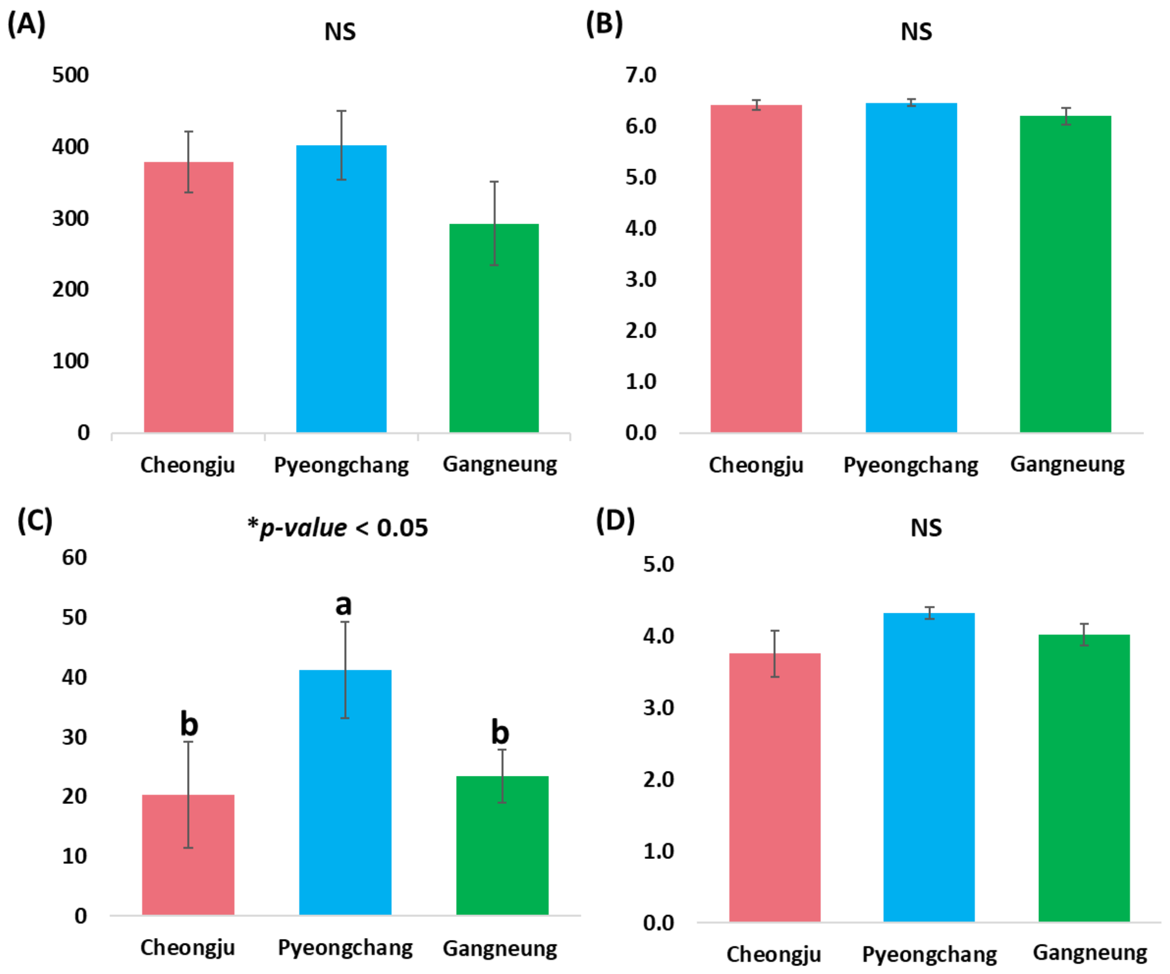

Supplemental Figure S8. Bacterial and fungal Shannon indices among three regions integrated both bulk and rhizosphere soil ASVs (A) Bacterial inversed Simpson index (B) Bacterial Shannon index (C) Fungal inversed Simpson index (D) Fungal Shannon index, Error bar represent standard error, ANOVA, NS: no significance

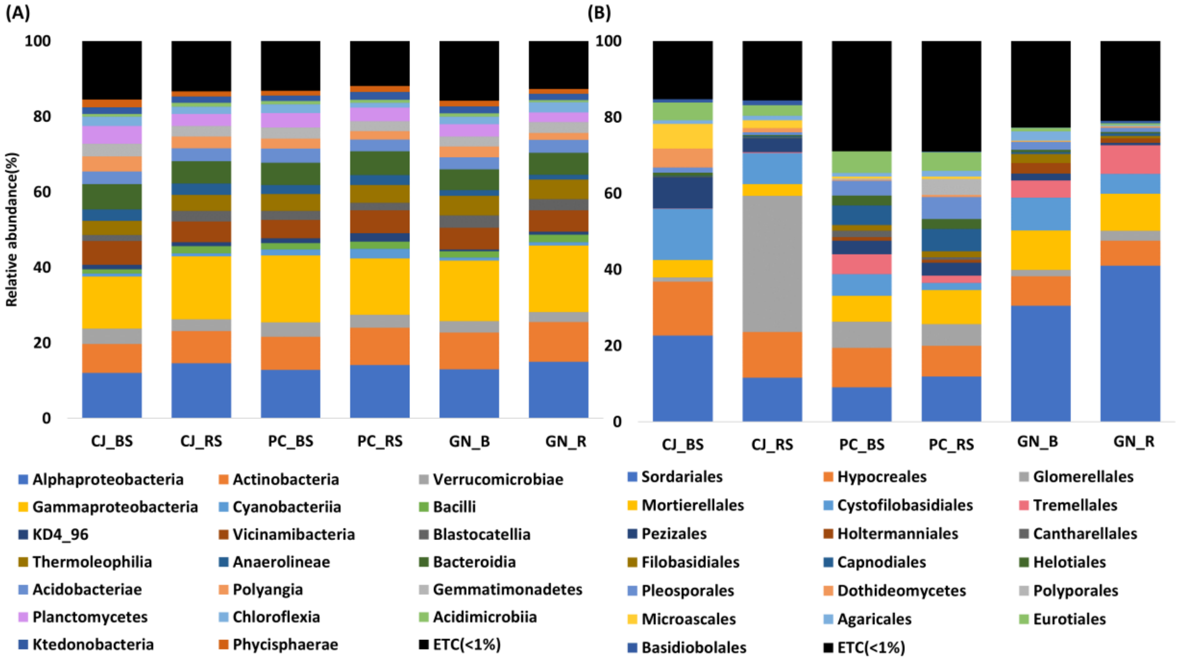

Supplemental Figure S9. Relative abundance of three regions bacterial (A) and fungal (B) microbiota. CJ_BS: Cheongju bulk soil, CJ_RS: Cheongju rhizosphere soil, PC_BS: Pyeongchang bulk soil, PC_RS: Pyeongchang rhizosphere soil, GN_BS: Gangneung bulk soil, GN_RS: Gangneung rhizosphere soil. Bacterial and fungal ASVs are described in Class and Order level, respectively

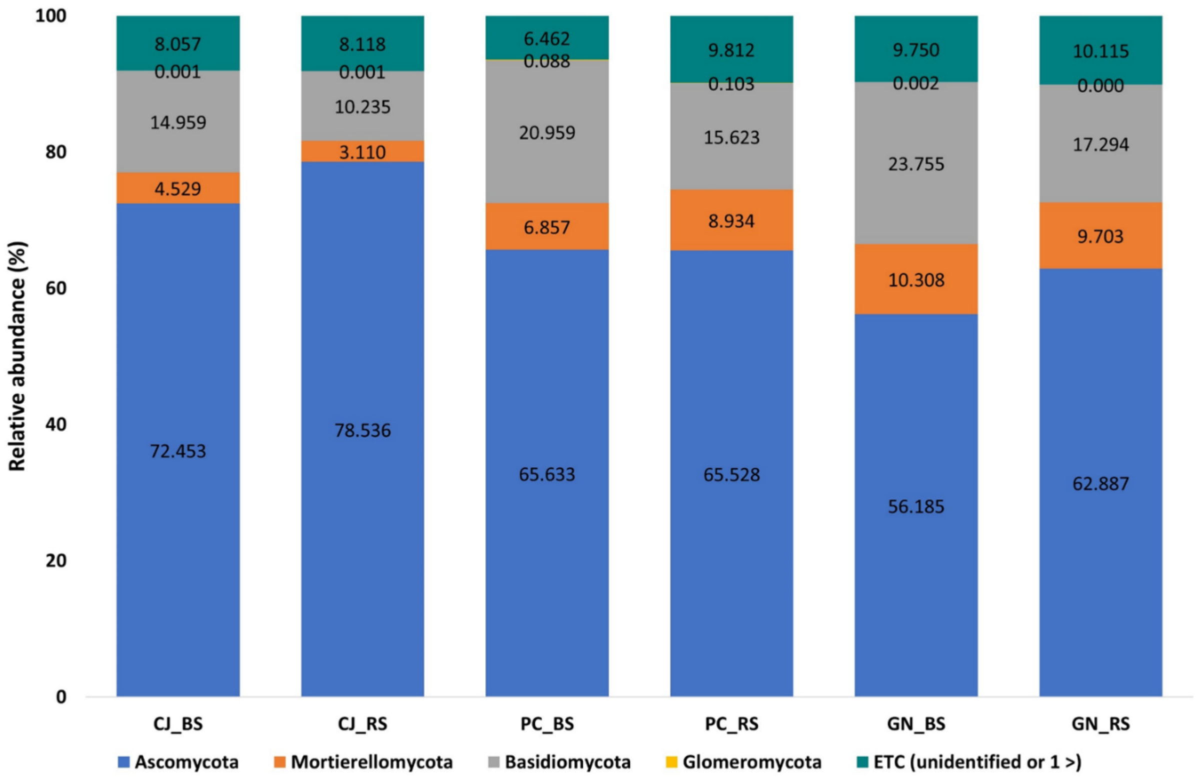

Supplemental Figure S10. Relative abundance of fungal phylum on three regions, CJ_BS: Cheongju bulk soil, CJ_RS: Cheongju rhizosphere soil, PC_BS: Pyeongchang bulk soil, PC_RS: Pyeongchang rhizosphere soil, GN_BS: Gangneung bulk soil, GN_RS: Gangneung rhizosphere soil

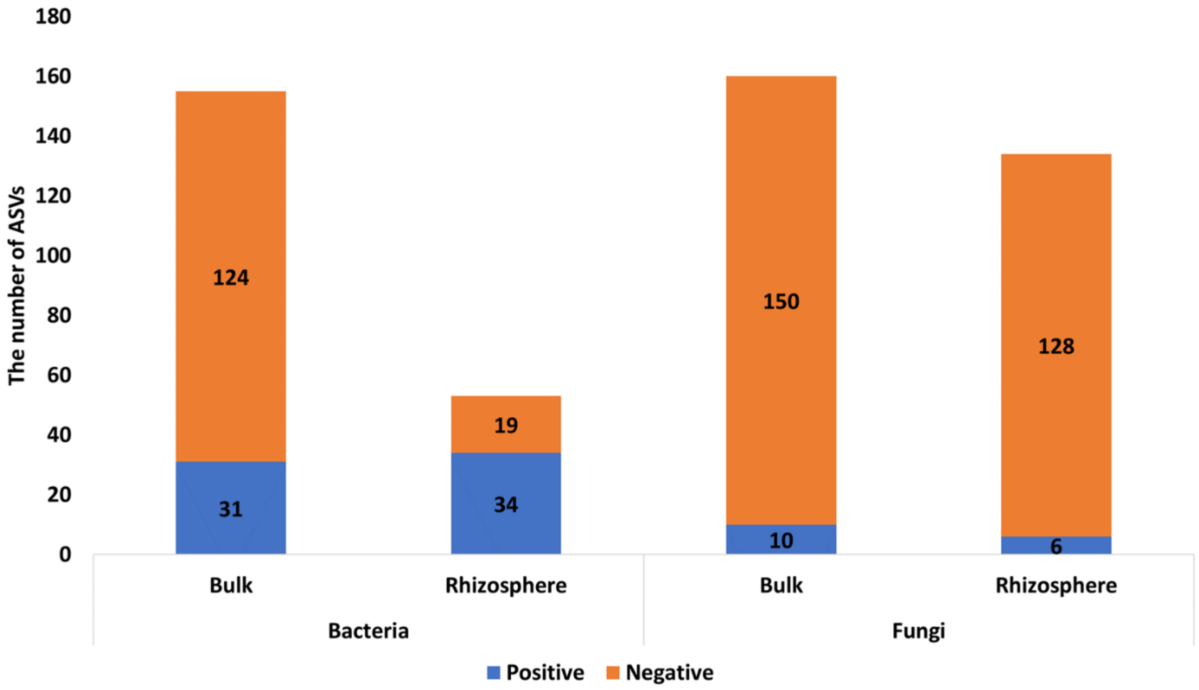

Supplemental Figure S11. The number of bacterial and fungal ASVs which positively or negatively correlated to potato yield, the number of ASVs were calculated of > 0.7 Spearman correlation coefficients
